# Perception of adults' smile esthetics among orthodontists, clinicians and laypeople

**DOI:** 10.1590/2176-9451.20.1.040-044.oar

**Published:** 2015

**Authors:** Enio Ribeiro Cotrim, Átila Valadares Vasconcelos, Ana Cristina Soares Santos Haddad, Sílvia Augusta Braga Reis

**Affiliations:** 1 Specialist in Orthodontics, Sérgio Feitosa Institute for Health Studies and Management (IES); 2 Assistant professor, IES; 3 Assistant professor, UMESP

**Keywords:** Orthodontics, Dental esthetics, Smile

## Abstract

**OBJECTIVE:**

Smile esthetics has become a major concern among patients and orthodontists. Therefore, the aim of this study was: (1) To highlight differences in perception of smile esthetics by clinicians, orthodontists and laypeople; (2) To assess factors such as lip thickness, smile height, color gradation, tooth size and crowding, and which are associated with smile unpleasantness.

**METHODS:**

To this end, edited photographs emphasizing the lower third of the face of 41 subjects were assessed by three groups (orthodontists, laypeople and clinicians) who graded the smiles from 1 to 9, highlighting the markers that evince smile unpleasantness. Kruskall-Wallis test supplemented by Bonferroni test was used to assess differences among groups. Additionally, the prevailing factors in smile unpleasantness were also described.

**RESULTS:**

There was no significant difference (P = 0.67) among groups rates. However, the groups highlighted different characteristics associated with smile unpleasantness. Orthodontists emphasized little gingival display, whereas laypeople emphasized disproportionate teeth and clinicians emphasized yellow teeth.

**CONCLUSION:**

Orthodontists, laypeople and clinicians similarly assess smile esthetics; however, noticing different characteristics. Thus, the orthodontist must be careful not to impose his own perception of smile esthetics.

## Abstract (pt)

**OBJETIVOS:**

a estética do sorriso tornou-se motivo de preocupação entre pacientes e ortodontistas. Diante disso, esse estudo objetivou (1) apontar as diferenças de percepção estética do sorriso entre grupos diferentes de avaliadores (clínicos, ortodontistas e leigos) e (2) avaliar entre fatores, como espessura de lábio, altura do sorriso, gradação de cor, tamanho dentário e apinhamento, quais estão implicados na desagradabilidade do sorriso.

**MÉTODOS:**

para tal, foram avaliados 41 indivíduos, com fotografias editadas, evidenciando o terço inferior da face. Essas fotografias foram avaliadas por três grupos (ortodontistas, leigos e clínicos), que graduaram os sorrisos com notas de 1 a 9 e apontaram marcadores que justificariam a desarmonia do sorriso. O teste de Kruskall-Wallis, complementado pelo teste de Bonferroni, foi empregado para verificação das diferenças entre os grupos de avaliadores. Além disso, os fatores mais prevalentes na desagradabilidade do sorriso foram descritos.

**RESULTADOS:**

não houve diferença significativa (p = 0,67) entre os grupos de avaliadores nas notas para avaliação do sorriso. Porém, os grupos apontaram características distintas, contribuindo para a desarmonia do sorriso. Para ortodontistas, a característica que mais contribuiu para a desarmonia do sorriso foi a pouca exposição gengival, enquanto, para os leigos, foram os dentes de tamanhos desproporcionais entre si, e, por último, dentes amarelados foram a característica mais observada pelos clínicos.

**CONCLUSÃO:**

concluiu-se que ortodontistas, leigos e clínicos, apesar de avaliarem similarmente a estética dos sorrisos, percebem características morfológicas diferentes. O ortodontista, portanto, deve ter cuidado sobre imposições de sua própria concepção de beleza do sorriso.

## INTRODUCTION

Smile esthetics has become a major concern among patients and orthodontists. It has been the main reason why patients seek orthodontic treatment.^1 ^The perception of beauty is associated with pleasure while seeing an object or a person, and while hearing a sound. For this reason, beauty is seen as a highly subjective feeling that results from individual factors such as sex, race, education and personal experiences, as well as social factors such as the environment and the media which has been increasingly responsible for globalizing the concept of beauty.^2 ^Assessing beauty is a highly subjective matter. Meanwhile, assessing patient's smile allows the clinician to see what needs to be done, what can be done and what should be accepted. Smile analysis includes assessing patient's smile arc, tooth and gingival display, presence of buccal corridor space (BCS), coincidence between facial and dental midlines, tooth proportionality, gingival esthetics, tooth color and occlusal plane inclination.^3^

A number of studies available in the literature have focused on smile geometric and objective analysis.^4^^-^ 8 Nevertheless, different factors might influence esthetic patterns, including culture. Furthermore, perception of esthetics varies considerably among individuals and is influenced by personal experiences as well as by the social environment.^9^

Thus, in addition to assessing patient's smile in geometrical and objective terms, it is also necessary to scientifically understand smile pleasantness from the point of view of laypeople, orthodontists and clinicians. Rodrigues et al^10 ^used printed photographs to assess smile attractiveness according to variations in esthetic norms evaluated by 20 laypeople. The authors concluded that variations in esthetic norms do not necessarily hinder perception of smile attractiveness, whereas diastema exerts strong negative influence on smile esthetics.

Schabel et al^11^ concluded that extremely unattractive smiles were characterized by great distance between the incisal edge of maxillary incisors and the lower lip, as well as by excessive smile height or insufficient smile width.

Sabherwal et al^12 ^compared the influence of skin and tooth color on smile attractiveness. The authors found that people with darker skin had lighter teeth in comparison to people with lighter skin; however, what most influenced the perception of white teeth was the color of gingiva and lips.

Dilalíbera et al^13 ^assessed the esthetic results of Class II patients subjected to corrective orthodontic therapy. Patients did not seem to be too concerned about the fact that facial angles and proportions did not coincide with what is mathematically proposed as esthetic, provided that these features were within the standards of normality accepted by them and established by society.

The literature has extensively covered the subject of smile in an objective manner; however, only a few studies have investigated the pleasant and unpleasant features of one's smile. With a view to discussing this issue and giving further contribution to the literature, this study aimed at:

» Highlighting the differences in perception of smile esthetics by clinicians, orthodontists and laypeople.
» Assessing factors such as lip thickness, smile height, color gradation, tooth size and crowding, which are associated with smile unpleasantness.

## MATERIAL AND METHODS

A total of 41 photographs of Brazilian, Caucasian patients (16 males and 25 females) aged between 18 and 56 years old (mean age of 37 years old) and with permanent dentition were analyzed. The photographs were taken from SENAI (Brazilian National Service of Industrial Training) students and employees. All subjects included in the sample signed an informed consent form. The research project was approved by local Institutional Review Board (protocol 2011/0199).

Image acquisition offered low risks to patients' well-being, since biosafety guidelines were strictly followed. Research volunteers were benefited from receiving orthodontic diagnosis and for being referred to treatment whenever necessary. Furthermore, the researcher was always willing to clarify potential doubts.

The following exclusion criteria were applied: Patients undergoing orthodontic treatment during data collection, and patients with craniofacial syndromes.

Standardized frontal facial photographs of patients' smile were used for analysis. All photographs were taken with Canon EOS Rebel XSI^(r)^ camera, flash Macro Ring Lite MR-14EX, Macro 100 sigma^(r)^ lens (Tokyo, Japan) and standardized with the same background. Patients were advised to keep natural head posture, remaining in the same posture they do in daily routine. In this research, patients were instructed to remain standing while looking ahead at the horizon. Photograph standardization was carried out in accordance with the parameters established by Reis et al.^14^

**Figure 1 -:**
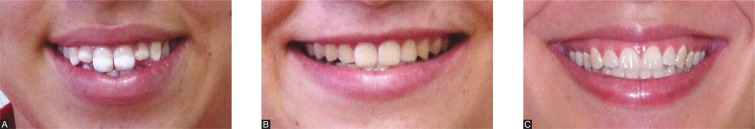
Frontal smile photographs representing each category: (A) esthetically unpleasant, (B) esthetically acceptable and (C) esthetically pleasant.

Frontal facial photographs of patients' smile were edited. In other words, they were cropped so as to evince the lower third of the face, particularly the smile. Examiners were asked to classify the photographs using scores from 1 to 9, as follows: esthetically unpleasant (scores 1, 2 or 3); esthetically acceptable (scores 4, 5 or 6) or esthetically pleasant (scores 7, 8 or 9) (Fig 1). Assessment was carried out by 5 orthodontists, 5 clinicians and 5 laypeople who also filled out a questionnaire so as to establish an association between smile unpleasantness and factors such as lip thickness, smile height, color gradation, teeth size and crowding.

Data were collected for descriptive statistics, highlighting the prevalence of pleasant, acceptable and unpleasant smiles as well as the mean scores attributed by each evaluator.

The scores attributed by the three groups of evaluators (orthodontists, clinicians and laypeople) were also submitted to Kruskall-Wallis statistical test supplemented by Bonferroni test so as to assess potential differences among groups. Additionally, the prevailing factors in smile unpleasantness were also described.

With a view to assessing intrarater agreement, ten facial photographs in frontal view were randomly selected and reassessed with a 30-day interval in between. Paired Student's t-test was used to assess systematic error. No significant difference was found between the first and second scores. Significance level was set at 5% (P > 0.05).

## RESULTS

Table 1 shows the values obtained by descriptive statistical analysis (mean, standard deviation and median) for subjective smile assessment.

**Table 1 -:** Descriptive statistics for subjective smile esthetics assessment.

	Orthodontists	Laypeople	Clinicians
Mean	4.78	4.48	4.89
SD	1.91	1.93	1.54
Median	5	4	5

Kruskall Wallis test did not reveal any difference among evaluators (orthodontists, laypeople and clinicians) (P = 0.67), whereas Bonferroni test found no significant differences between orthodontists and laypeople (P = 0.93), orthodontists and clinicians (P = 0.62) and between laypeople and clinicians (P = 0.29).

**Figure 2 -:**
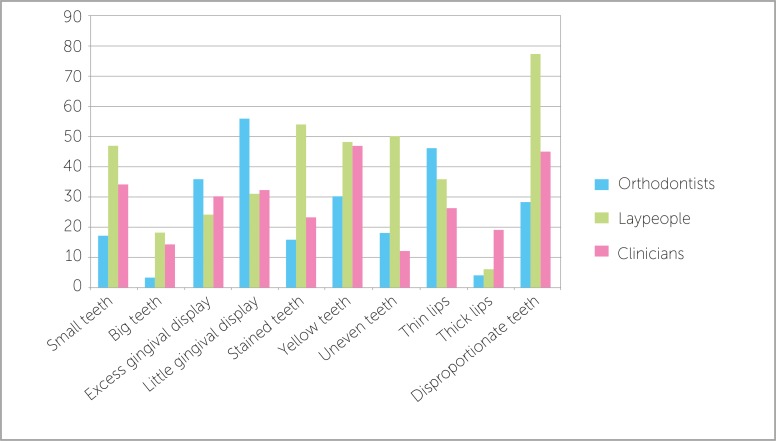
Different features of smile unpleasantness assessed by orthodontists, laypeople and clinicians.

Figure 2 shows the most prevalent factors observed in terms of smile unpleasantness, revealing that each group highlighted different features as being responsible for smile unpleasantness. Orthodontists emphasized little gingival display, whereas laypeople emphasized disproportionate teeth and clinicians emphasized stained teeth.

## DISCUSSION

In the present study, scores varied between 4 and 5. In other words, acceptable smiles were most prevalent in the sample studied. No differences were found among the scores attributed by each class of evaluators. However, each group assessed the sample from a different point of view, highlighting different features to classify the same smile as pleasant or unpleasant. Orthodontists emphasized the amount of gingival display and thin lips as the most prevalent features in unpleasant smile esthetics. Laypeople, on the other hand, emphasized stained, crowded, disproportional teeth as the features that most contribute to an unpleasant smile; whereas clinicians associated smile unpleasantness with stained, disproportional, small teeth. 

This means that beauty is subjective and, for this reason, establishing esthetic protocols for diagnosis and treatment planning based on orthodontists, clinicians and laypeople's perception might be a difficult task.

In all groups, thick lips and big teeth were less associated with smile unpleasantness (Fig 2), which suggests a cultural preference for proportionally big teeth and thick lips.

Only a few studies have been conducted to compare the opinion of different groups of evaluators about smile unpleasantness. Rodrigues et al^10^ demonstrated that smile assessment by laypeople differs from objective esthetic norms. Additionally, according to Van der Geld et al,^8 ^smiles characterized by total exposure of clinical crowns and gingival display not greater than 1 mm are considered more esthetic. In the present study, orthodontists evinced little gingival display as the most unpleasant feature. In the study by Malkinson et al,^15 ^smile esthetics was assessed by clinicians who found that excess gingival display influenced smile attractiveness and affected patient's attraction, reliability, intelligence and self-confidence. Machado et al^16 ^assessed progressive tooth wear and consequent asymmetry of anterior teeth. Their results agree with the present study, as they evince that tooth size discrepancy contributes to smile unpleasantness. 

The present study differs from other researches for identifying what characterizes smile unpleasantness instead of smile pleasantness.

The questionnaire applied in this study comprised pre-determined features of smile unpleasantness; however, other features could have been included, for instance, buccal corridor and curve of Spee. Ioi et al^17^ found that narrow or intermediate buccal corridors are considered more esthetic. Nevertheless, these features were not included in the present research due to being difficult to understand by laypeople.

This study evinced the importance of assessing patient's chief complaint and clinician's requirements so as to guide treatment planning. The orthodontist must be careful not to impose his own perception of smile esthetics.

## CONCLUSION

Based on the methods employed herein, it is reasonable to conclude that:

» The group conducting most strict smile assessment was that comprising laypeople, followed by orthodontists and clinicians. However, no statistical differences were found among groups.
» Laypeople were most concerned about disproportional teeth, whereas orthodontists evinced little gingival display and clinicians highlighted color gradation.
